# *Salmonella paratyphi* C: Genetic Divergence from *Salmonella choleraesuis* and Pathogenic Convergence with *Salmonella typhi*

**DOI:** 10.1371/journal.pone.0004510

**Published:** 2009-02-20

**Authors:** Wei-Qiao Liu, Ye Feng, Yan Wang, Qing-Hua Zou, Fang Chen, Ji-Tao Guo, Yi-Hong Peng, Yan Jin, Yong-Guo Li, Song-Nian Hu, Randal N. Johnston, Gui-Rong Liu, Shu-Lin Liu

**Affiliations:** 1 Genomics Research Center, Harbin Medical University, Harbin, China; 2 Microbiology and Infectious Diseases, University of Calgary, Calgary, Alberta, Canada; 3 JD Watson Institute of Genome Sciences, Zhejiang University, Hangzhou, China; 4 Beijing Institute of Genomics, Chinese Academy of Sciences, Beijing, China; 5 Department of Microbiology, Peking University Health Science Center, Beijing, China; 6 Depatment of Infectious Diseases, First Hospital, Harbin Medical University, Harbin, China; 7 Departments of Biochemistry and Molecular Biology, University of Calgary, Calgary, Alberta, Canada; University of Liverpool, United Kingdom

## Abstract

**Background:**

Although over 1400 *Salmonella* serovars cause usually self-limited gastroenteritis in humans, a few, e.g., *Salmonella typhi* and *S. paratyphi* C, cause typhoid, a potentially fatal systemic infection. It is not known whether the typhoid agents have evolved from a common ancestor (by divergent processes) or acquired similar pathogenic traits independently (by convergent processes). Comparison of different typhoid agents with non-typhoidal *Salmonella* lineages will provide excellent models for studies on how similar pathogens might have evolved.

**Methodologies/Principal Findings:**

We sequenced a strain of *S. paratyphi* C, RKS4594, and compared it with previously sequenced *Salmonella* strains. RKS4594 contains a chromosome of 4,833,080 bp and a plasmid of 55,414 bp. We predicted 4,640 intact coding sequences (4,578 in the chromosome and 62 in the plasmid) and 152 pseudogenes (149 in the chromosome and 3 in the plasmid). RKS4594 shares as many as 4346 of the 4,640 genes with a strain of *S. choleraesuis*, which is primarily a swine pathogen, but only 4008 genes with another human-adapted typhoid agent, *S. typhi*. Comparison of 3691 genes shared by all six sequenced *Salmonella* strains placed *S. paratyphi* C and *S. choleraesuis* together at one end, and *S. typhi* at the opposite end, of the phylogenetic tree, demonstrating separate ancestries of the human-adapted typhoid agents. *S. paratyphi* C seemed to have suffered enormous selection pressures during its adaptation to man as suggested by the differential nucleotide substitutions and different sets of pseudogenes, between *S. paratyphi* C and *S. choleraesuis*.

**Conclusions:**

*S. paratyphi* C does not share a common ancestor with other human-adapted typhoid agents, supporting the convergent evolution model of the typhoid agents. *S. paratyphi* C has diverged from a common ancestor with *S. choleraesuis* by accumulating genomic novelty during adaptation to man.

## Introduction

*Salmonella* are important human and animal pathogens [1], [2], and over 1400 serovars have the potential to cause human gastroenteritis, which is essentially a self-limited disease. However, a few *Salmonella* serovars, such as *S. typhi* and *S. paratyphi* A, B and C, elicit typhoid, which is a serious systemic infection with high mortality rates [3], [4]. *S. paratyphi* C as a typhoid agent [5]–[7] is not reported as frequently as *S. typhi* or *S. paratyphi* A or B, partly because this pathogen shares the antigenic formula 6,7:c:1,5 with *S. choleraesuis* and *S. typhisuis* and clinical identification of *Salmonella* isolates usually does not go beyond serotyping, although molecular methods are available to reliably distinguish *S. paratyphi* C from other Group C members [8]. As the gastroenteritis-causing and typhoidal *Salmonella* serovars are all closely related, sharing up to about 90% of their genetic contents [9]–[14], it is natural to ask how typhoid pathogenicity has developed in just a few of the thousands of *Salmonella* serovars. Specifically, are these similar pathogens the products of divergent (recent common ancestry) or convergent (common pathogenic traits incorporated into different genetic backgrounds) evolutionary processes?

Speculations have been made regarding these questions. The overwhelming genetic similarity (homologous genes having over 97% DNA sequence identity) and sharp pathogenic differences (self-limited local infections vs potentially fatal systemic infections) among the *Salmonella* serovars suggest horizontal acquisition of different pathogenic traits by these closely related bacteria. Whole genome comparisons at the physical map level reveal blocks of genomic insertions in different *Salmonella* lineages [15]–[22]. Genomic sequence comparisons identified 11–13% unique genes between *S. typhi* and *S. typhimurium* [9], [10], [12]. These results indicate that the *Salmonella* core genome provides a genetic framework for specific pathogenic determinants to be housed: acquisition of gastroenteritis- or typhoid-causing traits may “direct” the bacteria towards fundamentally distinct pathogens.

Among the human-adapted typhoid agents, comparison has been mostly focused on *S. typhi* and *S. paratyphi* A for elucidation of the genetic basis that might have led them to become fundamentally distinct pathogens. This pair of typhoid agents have greatly different sets of pseudogenes [11], suggesting separate immediate ancestries and thus favoring the convergence hypothesis of typhoid pathogenesis evolution. Later, the convergence model was again supported by a different approach, *i.e.*, a Bayesian changepoint model, which points out a high level of recombination between *S. typhi* and *S. paratyphi* A [23]. However, neither approach was conclusive about the evolution of the typhoid agents, largely due to the difficulty to have the divergent and convergent genomic events to be unambiguously distinguished. To reveal with greater confidence the genomic features common to the typhoid agents but not seen in non-typhoidal *Salmonella* pathogens for the elucidation of the genetic basis of the typhoid pathogenicity, we included additional typhoid agents as well as non-typhoidal salmonellae in the genomic comparisons. In this study, we determined the complete genome sequence of a strain of *S. paratyphi* C, RKS4594, and compared it with other published *Salmonella* genome sequences. Our analysis demonstrates that *S. paratyphi* C may have diverged from a common ancestor with *S. choleraesuis*, which is primarily a swine pathogen [13] but may also occasionally cause invasive infections in humans [24]–[27], relatively recently by adapting to a different niche and converged with *S. typhi* by accumulating genomic changes, including acquisition of genomic insertions and loss of certain genes.

## Results

### Overall features of *S. paratyphi* C and global comparisons with other *Salmonella* genomes

**Figure 1:**
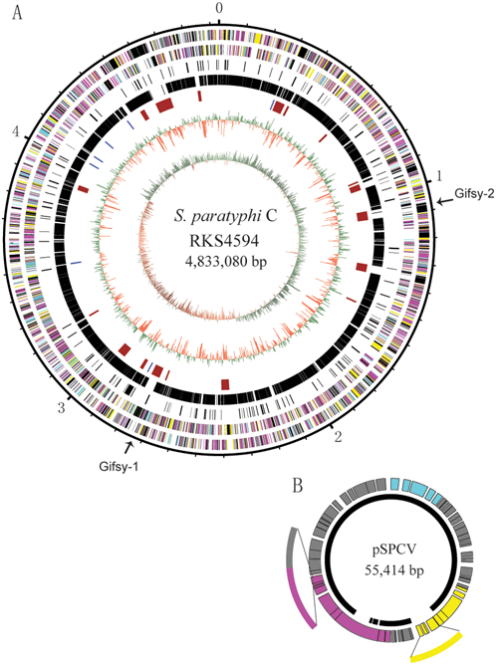
Map of the *S. paratyphi* C RKS4594 genome. (A) The chromosome. Circles range from 1 (outer circle) to 7 (inner circle): 1 and 2, genes on forward and reverse strand, respectively; 3, pseudogenes; 4, genes that are conserved among all six sequenced strains compared in this study; 5, rRNA operons (blue), and prophages and SPIs (brown); 6, G+C content, with values greater than average in green and smaller in red; and 7, GC skew (G−C/G+C), with values greater than zero in green and smaller in red. All genes displayed in circles 1 and 2 are colored by NCBI COG (Clusters of Orthologous Groups) function category: information storage and processing, cyan; cellular processes and signaling, yellow; metabolism, magenta; poorly characterized, black. The locations of two prophages, Gifsy2 and Gifsy 1, are indicated by arrows, which are recombination sites inverting a large chromosomal segment (ca. 1600 kb). (B) The virulence plasmid pSPCV. This plasmid comprises *spv* operon (cyan), *pef* operon (yellow), *tra* operon (magenta), and other regions (grey). The outermost arcs are additional regions of pSLT (a virulence plasmid from *S. typhimurium* LT2) compared with pSPCV. The inner black arc represents the conserved region of pKDSC50 (a virulence plasmid from *S. choleraesuis* SC-B67), pSPCV and pSLT. The gene content of pKDSC50, the most reduced of the three virulence plasmids, is virtually equal to the black arc.

*S. paratyphi* C RKS4594 was a clinical isolate and representative of a specific electrophoretic type, ET Pc-2, as determined by multi-locus electrophoresis; it was included in the set of the *Salmonella* Reference collection B strains (SARB49)[28]. This *S. paratyphi* C strain contains a chromosome of 4,833,080 bp and a plasmid of 55,414 bp (Fig. 1 and Table 1). We predicted 4,640 intact coding sequences (4,578 in the chromosome and 62 in the plasmid) and 152 pseudogenes (149 in the chromosome and 3 in the plasmid; Table S1). *S. paratyphi* C RKS4594 shares 3691 genes with all five previously sequenced *Salmonella* strains, including *S. typhi* CT18[9] and Ty2[12], *S. typhimurium* LT2 [10], *S. paratyphi* A ATCC9150[11], and *S. choleraesuis* SC-B67[13]; we define these genes as the *Salmonella* core genome (See details in Table S1). Between *S. paratyphi* C and each of the other five sequenced *Salmonella* strains, the actual numbers of shared genes differ greatly. For example, *S. paratyphi* C and *S. choleraesuis* share as many as 4346 genes, covering a total length of 4672 kb that accounts for 96.66% of the *S. paratyphi* C genome and 98.23% of the *S. choleraesuis* genome, with the differences being mostly clustered in prophages or *Salmonella* pathogenicity islands (SPIs) as large DNA segments. In contrast, *S. paratyphi* C RKS4594 and *S. typhi* CT18, both being human-adapted typhoid agents, share only 4008 genes, which account for 89.78% of the RKS4594 genome and 90.23% of the CT18 genome (similar percentages to the genes shared between *S. typhimurium* LT2 and *S. typhi* Ty2), with most differences being scattered throughout the genomes as small gene clusters. These data support the previous notions that *S. paratyphi* C is more closely related to *S. choleraesuis* than to *S. typhi* or *S. paratyphi* A [3]. We then looked further into the phylogenetic relationships of *S. paratyphi* C with five other sequenced *Salmonella* strains through systematic sequence comparisons.

**Table 1:** Summary of *S. paratyphi* C RKS4594 genome.

Features	Chromosome	Plasmid
Size, bp	4,833,080	55,414
G+C content, %	52.2	52.8
Coding density, %	88.5	82.3
ORFs (excluding pseudogenes):		
With assigned function	3,303	47
Unknown function	1,275	15
Total	4,578	62
Pseudogenes	149	3
Average ORF length, bp	887	634
rRNA operons	7	0
tRNAs	82	0

**Figure 2:**
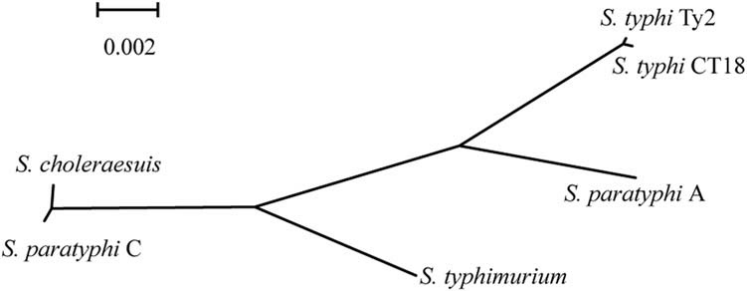
Phylogenetic tree for the six sequenced *Salmonella* strains, based on whole-genome sequences (all conserved regions among the six genomes are concatenated and aligned for tree construction).

We aligned sequences of common regions of the six *Salmonella* genomes and calculated genetic distances to estimate their relatedness. The resulting phylogenetic tree (Fig. 2) reveals a strikingly short genetic distance between *S. paratyphi* C and *S. choleraesuis*, indicating their very recent divergence, and a much greater distance from *S. paratyphi* C to *S. paratyphi* A, *S. typhi* or *S. typhimurium*. These data, again, strongly suggest that the typhoid-associated pathogenicity has evolved by convergent processes in different *Salmonella* genetic backgrounds.

### Comparison between *S. paratyphi* C and *S. choleraesuis*

The demonstrated recentness of the divergence between *S. paratyphi* C and *S. choleraesuis* suggests that we may still have an opportunity to track the evolutionary events that might have contributed to the evolution of a human-adapted typhoid agent. For this, we made systematic sequence comparisons between *S. paratyphi* C RKS4594 and *S. choleraesuis* SC-B67 [13].

We first focused on large genomic segments that differ between *S. paratyphi* C RKS4594 and *S. choleraesuis* SC-B67, as they are supposed to be laterally acquired and contain large numbers of genes that may have facilitated the pathogenic divergence process. Two regions, SPI-7 (SPC_4387–SPC_4471) and SPA-3-P2^SPC^ (SPC_0869–SPC_0908), are present in *S. paratyphi* C RKS4594 but absent from *S. choleraesuis* SC-B67. SPI-7 has only been found in *S. typhi*, *S. paratyphi* C and human-isolates of *S. dublin* [29]. In *S. typhi*, SPI-7 comprises four parts: type IVB pilus operon, SopE prophage, Vi biosynthetic operon, and a 15 kb phage-like segment [9], [29], whereas in many *S. paratyphi* C and *S. dublin* strains, only parts of type IVB pilus operon and Vi biosynthetic operon are present, with the SopE bacteriophage and the 15 kb phage-like segment being entirely absent [29], [30]. Despite its differences in structure among these bacteria, SPI-7 seems to have been acquired by *S. typhi* and *S. paratyphi* C fairly recently at around the same time, long after the emergence of *S. typhi* and *S. paratyphi* C. We made this speculation based on the fact that the sequence divergence of SPI-7 between *S. paratyphi* C and *S. typhi* (0.0006) is considerably lower than their chromosomal divergence (0.0179).

SPA-3-P2^SPC^ is highly similar to SPA-3-P2 of *S. paratyphi* A in sequence but has a different insertion site in the chromosome of *S. paratyphi* C RKS4594. Although SPI-7 and SPA-3-P2^SPC^ constitute the main genetic differences between *S. paratyphi* C and *S. choleraesuis*, they do not exist in all human-adapted typhoid agents (e.g., SPI-7 is not present in *S. paratyphi* A, SPA-3-P2 or SPA-3-P2^SPC^ is not present in *S. typhi*), suggesting that they are not necessarily a determinant for the typhoid pathogenesis. Other prophages and pathogenicity islands found in *S. paratyphi* C RKS4594 are summarized in Table S2.

We then made systematic sequence comparisons of the remaining parts of genomes between *S. paratyphi* C RKS4594 and *S. choleraesuis* SC-B67. These two strains have accumulated distinct sets of mutations, which is striking considering their very short divergence history. This is first reflected by their different sets of pseudogenes (Table S3). Although *S. paratyphi* C and *S. choleraesuis* have similar numbers of pseudogenes, with 152 in the former and 156 in the latter, only 55 are common to both. These findings may reflect distinct selection pressures on *S. paratyphi* C and *S. choleraesuis* when they were adapting to different niches.

The distinctness of accumulated mutations between *S. paratyphi* C and *S. choleraesuis* is also reflected by the exceptionally high non-synonymous (dN) over synonymous (dS) substitution ratio (dN/dS, ca. 0.62; Table 2), as compared to those between *S. paratyphi* C and *S. typhimurium*, *S. typhi* or *S. paratyphi* A, which are in the range of 0.13–0.15 (Table 2). The two sequenced *S. typhi* strains also have a high dN/dS ratio (Table 2), but the mechanisms might be different.

**Table 2:** dN and dS values in pairs of compared genomes.

Genomes compared	dN	dS	dN/dS
SPC vs SC	0.00131(±0.00697)	0.00209(±0.00940)	0.62453
SPC vs STM	0.00459(±0.03428)	0.03416(±0.14710)	0.13451
SPC vs CT18	0.00642(±0.03156)	0.04564(±0.14594)	0.14074
SPC vs Ty2	0.00641(±0.03156)	0.04568(±0.14611)	0.14029
SPC vs SPA	0.00726(±0.04701)	0.04762(±0.18746)	0.15252
CT18 vs Ty2	0.00016(±0.00329)	0.00029(±0.00601)	0.57240

**Footnote:** SPC, *S. paratyphi* C RKS4594; SC, *S. choleraesuis* SC-B67; STM, *S. typhimurium* LT2; Ty2, *S. typhi* Ty2; CT18, *S. typhi* CT18; SPA, *S. paratyphi* A ATCC9150.

To reveal the actual nucleotide substitutions that would lead to amino acid changes, we aligned the sequences coding for 3238 proteins common to all six *Salmonella* genomes compared and identified 2335 amino acids that are different between *S. paratyphi* C and *S. choleraesuis*. Since as many as 2222 of the 2335 amino acids are identical in *S. typhi*, *S. paratyphi* A and *S. typhimurium*, we assumed these amino acids to be the state in the ancestors of *S. paratyphi* C, *S. choleraesuis*, *S. typhi*, *S. paratyphi* A and *S. typhimurium*. Of these 2222 amino acids that are common to *S. typhi*, *S. paratyphi* A and *S. typhimurium*, *S. paratyphi* C has 1147 (the other 1075 being different from those in *S. typhi*, *S. paratyphi* A, *S. typhimurium* and *S. choleraesuis*) and *S. choleraesuis* has 1028 (the other 1194 being different from those in *S. typhi*, *S. paratyphi* A, *S. typhimurium* and *S. paratyphi* C), suggesting differential selection pressures to “force” *S. paratyphi* C and *S. choleraesuis* to have these distinct sets of particular amino acids selected for their eventual adaptation to different niches. Of special interest is a list of nine amino acids in *S. paratyphi* C RKS4594 that are different from their counterparts in *S. choleraesuis* SC-B67 but identical to those of either *S. typhi* or *S. paratyphi* A (Table S4), possibly reflecting a need of these particular amino acids by the human-adapted *Salmonella* lineages for dwelling in the host. These features again strongly indicate *S. paratyphi* C as an ideal model in studies to elucidate the molecular mechanisms of human adaptation during the evolution of a typhoid agent from its host-generalist ancestor.

### Common gains and losses of genes among the sequenced typhoid agents

We examined possible common gains and losses among the sequenced human-adapted typhoidal strains, relative to *S. typhimurium* LT2. Systematic comparisons of these typhoidal strains with *S. typhimurium* LT2 did not lead to the identification of genes common only to the human-adapted typhoidal strains. This raises two possibilities: (i) different *Salmonella* typhoid agents might have acquired different typhoid-causing traits, as suggested by the large number of genes common to *S. typhi* and *S. paratyphi* A [23] but not to *S. paratyphi* C, or SPI7 common to *S. typhi* and *S. paratyphi* C but not to *S. paratyphi* A; and (ii) many *Salmonella* serovars might carry genes that would participate in typhoid pathogenesis only in a small number of serovars due to the acquisition (or activation) or loss (or inactivation) of other genes. Both scenarios favor the convergence evolution model of the typhoid agents, implicating that the immediate ancestors of the extant human-adapted *Salmonella* lineages acquired the typhoid-causing traits independently and then converged under the same host pressure to become clinically similar pathogens.

On the other hand, we found that a total of 24 genes were either absent or inactivated in the sequenced *S. typhi*, *S. paratyphi* A and *S. paratyphi* C strains relative to *S. typhimurium* LT2 (Table 3), which suggests that these functions are not required for human infection. Of special interest are genes encoding fimbriae, as fimbriae have long been known to constitute a “signature” for *Salmonella* serovars [31], [32]. More importantly, *Salmonella* fimbriae are known to be involved in infections and may play a role in host determination [33]. Although human-adapted typhoid agents possess special repertories of fimbrial genes that are involved in the bacterial infection process in humans [32], the inability of these bacteria to infect other host may be accounted for by loss of certain fimbial genes. We found that three fimbial genes, *safC*, *bcfC* and *stfD*, are pseudogenes in the sequenced *S. typhi*, *S. paratyphi* A and *S. paratyphi* C strains, and one fimbial gene, *stj*, is entirely absent in the sequenced *S. typhi*, *S. paratyphi* A and *S. paratyphi* C strains; these four fimbial genes are present and intact in *S. typhimurium* LT2.

**Table 3:** Deletion and pseudogene formation in the four human-adapted typhoidal strains.

Locus_tag	Symbol	Product	Category
SPC_0797	*slrP*	leucine-rich repeat protein	1
SPC_1513	*mglA*	galactose (methyl-galactoside) transport protein	1
SPC_1647	*sopA*	secreted effector protein	1
SPC_1757	*fliB*	N-methylation of lysine residues in flagellin	1
SPC_2542	*fhuE*	outer membrane receptor for ferric iron uptake	1
SPC_4172	*-*	putative permease of the Na+:galactoside symporter family	1
SPC_0673	*ybeS*	putative molecular chaperone, DnaJ family	2
SPC_0675	*ybeV*	putative molecular chaperone, DnaJ family	2
SPC_0760	*-*	transcriptional regulator, lysR family	2
SPC_0859	*-*	putative inner membrane protein	2
SPC_1396	*yfbK*	putative von Willebrand factor, vWF type A domain	2
SPC_1703	*-*	putative endoprotease	2
SPC_2105	*-*	putative inner membrane protein	2
SPC_2378	*ydiS*	flavoprotein	2
SPC_2702	*-*	Gifsy-2 prophage host specificity protein J	2
SPC_3146	*-*	putative mannitol dehydrogenase	2
SPC_3591	*rtcR*	sigma N-dependent regulator of rtcBA expression	2
SPC_2077	*-*	putative methyl-accepting chemotaxis protein	3
SPC_2458	*-*	putative Methyl-accepting chemotaxis protein	3
SPC_2232	*dmsB*	anaerobic dimethyl sulfoxide reductase chain B	3
SPC_0311	*safC*	fimbrial operon protein	3
SPC_0025	*bcfC*	fimbrial operon protein	3
SPC_0213	*stfD*	fimbrial operon protein	3
putative deletion	*stj*	fimbrial operon protein	3

Note: we divide the presumably lost genes, relative to *S. typhimurium* LT2, into three categories: 1, they are all pseudogenes in the four typhoidal strains; 2, they are pseudogenes in *S. paratyphi* C but entirely absent in *S. typhi* and *S. paratyphi* A; and 3, other genes in the same or a similar pathway are either pseudogenes or absent.

### Chromosomal rearrangement mediated by Gifsy sequences

Previously, we reported that physical balance of the bacterial chromosome between replication origin, *oriC*, and terminus, *terC*, affects growth rate in *S. typhi* and therefore may influence the competition capability of the bacteria in nature [34]. Unlike *S. typhimurium* and *S. paratyphi* A, which have well balanced and very stable genome structures [20], [35], *S. paratyphi* C and *S. typhi* both have less optimally balanced genomes and so have undergone frequent rearrangements [18], [19], [21]. Most often, chromosomal rearrangements occur through recombination between homologous sites such as *rrn* genes [18] or IS*200* [36]. However, as *S. paratyphi* C does not have IS*200* (See Table S1), most genomic rearrangements among wild type strains of *S. paratyphi* C are mediated by *rrn* genes [21], with an important exception as detailed below.

**Figure 3:**
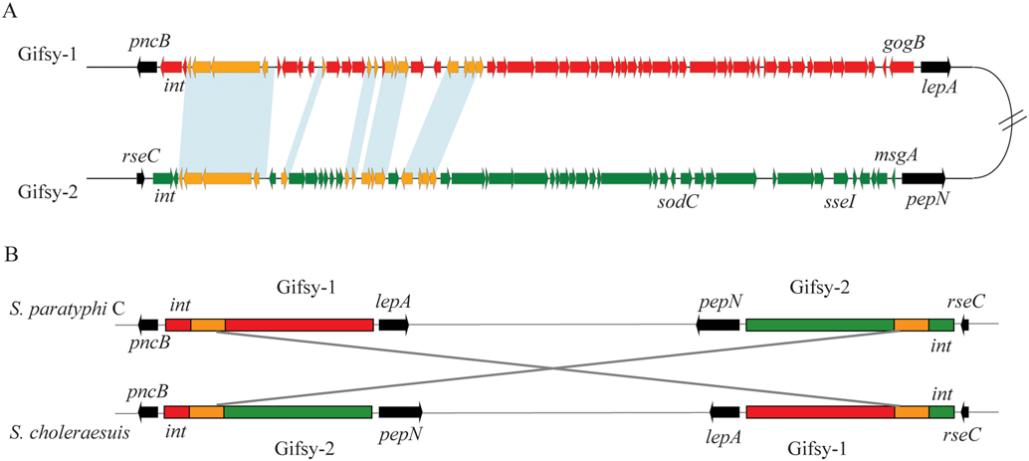
Chromosomal rearrangement mediated by Gifsy-1 and Gifsy-2. (A) Alignment of Gifsy-1 and Gisfy-2 in *S. paratyphi* C. Common genes in Gifsy-1 and Gifsy-2 are colored in yellow, with the remaining genes of Gifsy-1 being colored in red and those of Gifsy-2 in green; conserved genes adjacent to Gifsy-1 and 2 are in black. The blue shade indicates identity >90% between Gifsy-1 and Gifsy-2. (B) Chromosomal comparison showing the relative inversion between *S. paratyphi* C and *S. choleraesuis*. The scheme is color-coded as above and shows that the broad ranges of homologous regions between Gifsy-1 and Gifsy-2 instead of integrases mediate the chromosomal rearrangement.

In RKS4594, *oriC* is located at 4016 kb and *terC* is around 2256 kb from *thrL*. As the genome size is 4833 kb, the balance is 249° clockwise and 131° counterclockwise between *oriC* and *terC*, which is far off the 180° balanced status. Through comparison with *S. typhimurium* LT2, an inversion of about 1600 kb was found between homologous regions of prophages Gifsy-1 and Gifsy-2 (Figure 3), which was confirmed by physical mapping [21]. To our knowledge, it is the first report of prophage mediated chromosomal rearrangement in *Salmonella*.

### Virulence plasmid

*S. paratyphi* C RKS4594 contains a plasmid, pSPCV, with very high sequence identity with the virulence plasmids pSLT (*S. typhimurium* LT2) [10] and pKDSC50 (*S. choleraesuis* SC-B67) [13]. The three plasmids have decreasing sizes from pSLT (93.9 kb) to pSPCV (55.4 kb) and to pKDSC50 (49.6 kb), suggesting a gradual degradation process to shed unnecessary genes during evolution. All three *Salmonella* plasmids contain operons *spv*, *pef* and *tra*. The *spv* operon is conserved among all *Salmonella* virulence plasmids that have been characterized to date [37] and is proven to be required for the systemic phase of the infection in their host [38].

The genes *pefABCD* in the *pef* operon (plasmid-encoded fimbriae) are conserved among the three plasmids. However, the downstream region, i.e., between *pefD* and the *repA* loci, shows remarkable variability between pSLT and pSPCV, and is entirely absent from pKDSC50 (see Fig. 1b). Within this region of pSLT, two genes, *srgA* (PSLT011, encoding thiol∶disulphide oxidoreductase) and *srgB* (PSLT010, encoding a putative outer membrane protein), have significant homology to two genes in SPI-10 of both *S. typhi* and *S. paratyphi* A. As most *S. typhi* and *S. paratyphi* A strains do not have virulence plasmids, it was once speculated that the two genes might partly complement the functions of the virulence plasmids [39]. However, because the *srgA* counterparts in *S. typhi* CT18, Ty2 and *S. paratyphi* A ATCC9150 have become inactivated by a frameshift mutation, and because the genes *srgAB* are either degraded or deleted in pSPCV and pKDSC50, it is obvious that the two genes are not involved in virulence or other key biological activities.

The *tra* operon is responsible for conjugative transfer of the plasmid. pSLT has intact *tra* operon and is self-transmissible [40]. In pSPCV and pKDSC50, the operon is in the process of degradation.

We examined the divergence calculated from conserved regions of the three plasmids. The divergence between pSPCV and pSLT and that between pSPCV and pKDSC50 is 0.0176 and 0.0020, respectively, both of which are fairly consistent with the divergence levels of the chromosomes between *S. paratyphi* C and *S. typhimurium* LT2 (0.0117) and between *S. paratyphi* C and *S. choleraesuis* (0.0011). The consistence of divergence levels between the chromosomal and plasmid sequences strongly suggests the vertical inheritance nature of the plasmids.

## Discussion

Analysis of the *S. paratyphi* C genome has revealed to us new facts about the genetic divergence of *Salmonella* pathogens and helped clarify the phylogenetic relationships among the human-adapted typhoid agents and other *Salmonella* lineages. This work will also significantly facilitate the studies of pathogenic divergence of *Salmonella* as a whole and, especially, the Group C *Salmonella* lineages bearing the common antigenic formula 6,7:c:1,5, including *S. paratyphi* C, *S. choleraesuis* and *S. typhisuis* [41]. The highly similar genomic constructions between *S. paratyphi* C and *S. choleraesuis* [13] and their distinct pathogenic features [5], [42] make them excellent models for studies of *Salmonella* host adaptation and pathogenic divergence. Our results strongly suggest that the two lineages had a common immediate ancestor and that they diverged fairly recently and provide further evidence about the closer relatedness between *S. paratyphi* C and *S. choleraesuis* than either to *S. typhi* or *S. paratyphi* A [3]. Perhaps an occasional invasion of and gradual adaptation to human body caused a branch of the ancestor to become settled in the new niche. During this process, favorable changes of nucleotides/amino acids may have been quickly selected and accumulated to facilitate the host shift, as reflected by the greater dN than dS substitutions between *S. paratyphi* C and *S. choleraesuis*.

Usually, when a bacterial lineage begins to diverge from the ancestor, dN may transiently be greater than dS among members of the same diverging lineage due to the nature of genetic codons (changes in the first two of the three nucleotides in a codon tends to cause dN). For example, *S. typhi* may have diverged from its ancestor for no more than fifty thousand years [43] and individual strains still have relatively high dN/dS values as seen between CT18 and Ty2 (See Table 2). Then as deleterious mutations (i.e., many dNs) are eventually purged [44], dS would gradually exceed dN. Therefore, the ratio of dN/dS may in a way reflect evolutionary distances among a certain range of closely related bacteria living in the same kind of niche, e.g., independent isolates of *S. typhi* that dwell only in the human body; in such cases, more closely related strains may have greater dN/dS values than more distantly related ones and the dN/dS ratio may then decrease with time. We speculate that the scenario may be different, however, in bacteria that are closely related but do not dwell in the same niche, such as *S. paratyphi* C and *S. choleraesuis*, in which the dN/dS ratio may remain relatively high for long evolutionary times due to the potential benefits brought to the bacteria by the non-synonymous nucleotide substitutions.

Although several lines of evidence, especially those presented in this paper, support the convergence evolution model of the human-adapted typhoid agents [11], [23], genes directly contributing to the typhoid phenotypes remain to be identified. In this study, we compared the genomes of the human-adapted typhoidal strains (*S. typhi* CT18 and Ty2, *S. paratyphi* A ACTT9150 and *S. paratyphi* C RKS4594) with those of *S. typhimurium* LT2 and *S. choleraesuis* SC-B67 to attempt identifying typhoid-associated genes. However, we did not obtain a significant list of genes present in the former but absent in the latter. This might be because *S. typhimurium*, though causing gastroenteritis in humans, does have genes to cause typhoid-like disease in mice and some of the genes might be related to those in the human-adapted typhoid agents. Additionally, *S. choleraesuis*, having a narrow host range and causing invasive infections in humans [24]–[27], can cause swine paratyphoid [45]. Therefore, all six sequenced *Salmonella* strains compared in this study have the potential of causing typhoid-like diseases in humans or animals, so none of them could be used as a real “typhoid-free” reference for comparison to identify typhoid-associated genes. As a result, the genomic sequence of a *Salmonella* lineage that does not cause typhoid-like disease in any host, such as *S. pullorum*, is desired in studies for further narrowing down the typhoid determinants. On the other hand, the large number of degraded genes (pseudogenes) and the distinct set of selected amino acids (dN) identified in the *S. paratyphi* C genome through this study will provide a guide in studies for the elucidation of the genetic basis for host adaptation of this pathogen to humans.

### Conclusions

*S. paratyphi* C does not share a common ancestor with other human-adapted typhoid agents, supporting the convergent model of the evolution of the typhoid agents. *S. paratyphi* C has diverged from a common ancestor with *S. choleraesuis* by accumulating genomic novelty during adaptation to man.

## Materials and Methods

### Sequencing

The genome sequence of *S. paratyphi* C RKS4594 was determined by dye terminator chemistry on Megabace1000 and ABI3730 automated sequencers, with DNA clones from several pUC18 genomic shotgun libraries (insert sizes ranging from1.5 to 4.0 kb). The Phred/Phrap/Consed package was used for quality assessment and sequence assembly. Gaps were filled by PCR amplification and primer walking methods. Ambiguous areas were re-sequenced and the assembly was verified by a physical map; the final sequence reached accuracy over 99.99%.

### Annotation

Gene prediction was performed by use of GLIMMER3 and by comparison with the annotated genes from five available *Salmonella* genomes, i.e., *S. typhimurium* LT2, *S. choleraesuis* SC-B67, *S. paratyphi* A ATCC9150, and *S. typhi* CT18 and Ty2. Intergenic regions were searched against NCBI non-redundant libraries for potential genes. The function of all coding sequences was further investigated by searching against InterPro database. Genes that contains insertion, deletion or mutation to a stop codon compared with those known *Salmonella* genes were categorized as pseudogenes. Transfer RNA genes were predicted with tRNAscan-SE, and ribosomal RNA genes were predicted by similarity to other *Salmonella* rRNA genes.

### Comparative and phylogenetic analysis

Whole genome alignment was made by use of MAUVE and MUMmer program. Phylogenetic tree construction was done with PHYLIP 3.6 package. Nucleotide divergence in this article was defined as the number of mismatch bases divided by that of comparable bases after pairwise alignment made by CLUSTALW. dN/dS values were calculated with Yn00 program in PAML 3.15 package.

### URLs

The Phred/Phrap/Consed package is available at http://www.phrap.org/phredphrapconsed.html. GLIMMER3 is available at http://www.cbcb.umd.edu/software/glimmer/. NCBI non-redundant libraries is available at ftp://ftp.ncbi.nih.gov/blast/db/. InterPro database is available at http://www.ebi.ac.uk/interpro/. tRNAscan-SE is available at http://lowelab.ucsc.edu/tRNAscan-SE/. MAUVE program is available at http://gel.ahabs.wisc.edu/mauve/. MUMmer program is available at http://mummer.sourceforge.net/. PHYLIP 3.6 package is available at http://evolution.genetics.washington.edu/phylip.html. CLUSTALW program is available at http://www.ebi.ac.uk/Tools/clustalw/. PAML 3.15 package is available at http://abacus.gene.ucl.ac.uk/software/paml.html.

### Accession numbers

Genbank: *S. typhimurium* LT2 [NC_003197]; *S. choleraesuis* SC-B67 [NC_006905]; *S. paratyphi* A ATCC9150 [NC_006511]; *S. typhi* CT18 [NC_003198]; *S. typhi* Ty2 [NC_004631]; *S. paratyphi* C RKS4594 chromosome [CP000857]; *S. paratyphi* C RKS4594 plasmid pSPCV [CP000858].

## Supporting Information

Table S1 Genome annotation
(1.23 MB XLS)

Tables S2 and S3 (0.32 MB DOC)

Table S4 (0.02 MB XLS)
