# Supplementary material for: Salmonella paratyphi C: Genetic Divergence from Salmonella choleraesuis and Pathogenic Convergence with Salmonella typhi
Source: PLoS One. 2009 Feb 20;4(2):e4510. doi: 10.1371/journal.pone.0004510 (PMC2640428; doi:10.1371/journal.pone.0004510)
Supplement: Tables S2 and S3 — (0.32 MB DOC) [file pone.0004510.s002.doc]

**Supplementary tables**

**S Table 1.** Annotation of the *S. paratyphi* C RKS4594 genome and its comparisons with the other five sequenced *Salmonella* genomes.

This table is too large to be put here. See the attached Excel file.

**S Table 2.** SPIs and prophages in *S. paratyphi* C RKS4594.

| **Name** | **Location** | **No. of genes** | **Comments** |
| --- | --- | --- | --- |
| SPI-1 | SPC_2903-SPC_2950 | 48 | Conserved among all six sequenced strains |
| SPI-2 | (t)SPC_t038-SPC_2350 | 44 | Conserved among all six sequenced strains |
| SPI-3 | (t)SPC_t059-SPC_3854 | 22 | Conserved among all six sequenced strains |
| SPI-4 | SPC_4321-SPC_4326 | 6 | Conserved among all six sequenced strains |
| SPI-5 | SPC_2656-SPC_t042(t) | 8 | Conserved among all six sequenced strains |
| SPI-6 | (t)SPC_t004-SPC_0319 | 45 | SPI-6 in *S. typhimurium* and *S. paratyphi* C is remnant compared to their counterparts in *S. cholaeraesuis*, *S paratyphi* A and *S. typhi*. |
| SPI-7 | SPC_4387-SPC_4471(t) | 84 | Only present in *S. paratyphi* C and *S. typhi* |
| SPI-9 | SPC_2799-SPC_2802 | 16 | Conserved among all six sequenced strains |
| SPI-12 | SPC_1465-SPC_t027(t) | 7 | Showing a gradual degradation process: from *S. typhimurium* to *S. paratyphi* C, to *S. choleraesuis*, to *S. typhi* and to *S. paratyphi* A |
| SPI-13 | (t)SPC_t051-SPC_3190 | 7 | Present in *S. typhimurium*, *S. paratyphi* C and *S. choleraesuis* |
| SPI-14 | SPC_0852-SPC_0858 | 7 | Present in *S. typhimurium*, *S. paratyphi* C and *S. choleraesuis* |
| SPI-16 | (t)SPC_t006-SPC_0578 | 11 | Conserved among the six sequenced strains |
| Gifsy-1 | SPC_1007-SPC_1065 | 59 | A virulent prophage that, together with Gifsy-2, mediates chromosomal rearrangement in *S. paratyphi* C; this prophage is also present in *S. typhimurium* and *S. choleraesuis* |
| Gifsy-2 | SPC_2693-SPC_2746 | 54 | A virulent prophage that, together with Gifsy-1, mediates chromosomal rearrangement in *S. paratyphi* C; this prophage is also present in *S. typhimurium*, *S. choleraesuis* and *S. typhi* |
| SPC-phage-1 | SPC_1264-SPC_t026(t) | 50 | A prophage, which is similar to SPA-1^SC^ (SC0324-SC0378) in *S. choleraesuis* but has a different insertion site |
| SPI-SPC-SC | (t)SPC_t079-SPC_4630 | 12 | *S. paratyphi* C and *S. choleraesuis* both have this SPI; in the same insertion site, *S. typhi* and *S. paratyphi* A have SPI-10 |
| Phage SPA-1 | (t)SPC_t005-SPC_0345 | 10 | This segment in *S. typhimurium* and *S. paratyphi* C is remnant of a prophage in *S. paratyphi* A and *S. choleraesuis* |
| SPA-3-P2^spc^ | SPC_0869-SPC_0908 | 40 | SPA-3-P2^spc^ in *S. paratyphi* C is very similar to SPA-3-P2 in *S. paratyphi* A but has a different insertion site. |

Note: (t) indicates tRNA. In this table, *S. typhimurium* refer to the strain LT2; *S. paratyphi* C refers to the strain RKS4594; *S. choleraesuis* refers to the strain SC-B67; *S. paratyphi* A refers to the strain ATCC9150; *S. typhi* refers to both strains CT18 and Ty2.

**S Table 3.** Comparison of pseudogenes from *S. paratyphi* C and *S. choleraesuis*.

*(A) List of pseudogenes common for S. paratyphi C and S. choleraesuis*.

| *S. paratyphi* C | *S. choleraesuis* | symbol | product |
| --- | --- | --- | --- |
| SPC_0025 | SCPS38 | *bcfC* | fimbrial usher |
| SPC_0037 | SCPS58 | *-* | putative arylsulfatase |
| SPC_0040 | SCPS125 | *-* | putative arylsulfatase |
| SPC_0109 | SC0096_SC0097 | *araA* | L-arabinose isomerase |
| SPC_0336 | SCPS67 | *-* | putative HSP70 class molecular chaperone |
| SPC_0630 | SCPS97 | *rna* | ribonuclease I precursor |
| SPC_0675 | SCPS100 | *ybeV* | putative molecular chaperone, DnaJ family |
| SPC_0760 | SCPS134 | *-* | transcriptional regulator, lysR family |
| SPC_0822 | SCPS3 | *-* | putative SAM-dependent methyltransferase |
| SPC_0859 | SCPS5 | *-* | putative inner membrane protein |
| SPC_0984 | SCPS136 | *-* | putative recombination protein |
| SPC_1195 | SC2460_SC2461 | *eutN* | putative detox protein |
| SPC_1513 | SCPS36 | *mglA* | galactose (methyl-galactoside) transport protein |
| SPC_1546 | SC2172 | *-* | putative lipoprotein |
| SPC_1589 | SCPS35 | *-* | putative MFS family transport protein |
| SPC_1647 | SC2076 | *sopA* | secreted effector protein |
| SPC_1703 | SCPS141 | *-* | putative endoprotease |
| SPC_1726 | SCPS34 | *yedI* | putative inner membrane protein |
| SPC_1806 | SCPS107 | *cutC* | putative copper homeostasis protein |
| SPC_1816 | SCPS33 | *yebB* | putative periplasmic protein |
| SPC_2054 | SCPS31 | *hemA* | glutamyl-tRNA reductase |
| SPC_2098 | SCPS138 | *-* | hypothetical protein |
| SPC_2105 | SCPS137 | *-* | putative inner membrane protein |
| SPC_2147 | SCPS25 | *-* | putative regulatory protein |
| SPC_2168 | SCPS22 | *-* | putative molybdopterin oxidoreductases |
| SPC_2232 | SC1514 | *dmsB* | anaerobic dimethyl sulfoxide reductase chain B |
| SPC_2250 | SCPS18 | *-* | putative membrane protein |
| SPC_2257 | SC1491_SC1492 | *-* | hypothetical protein |
| SPC_2353 | SC1396_SC1397 | *lppB* | major outer membrane lipoprotein |
| SPC_2401 | SCPS16 | *-* | hypothetical protein |
| SPC_2475 | 1381399_1381245 bp | *-* | hypothetical protein |
| SPC_2496 | 1365333_1365158 bp | *-* | hypothetical protein |
| SPC_2603 | SCPS103 | *-* | putative ACR protein |
| SPC_2702 | SCPS10 | *-* | Gifsy-2 prophage host specificity protein J |
| SPC_2799 | SC2689_SC2692 | *-* | large repetitive protein |
| SPC_3018 | SCPS52 | *-* | putative d-glucarate dehydratase |
| SPC_3096 | SC2976 | *-* | putative inner membrane protein |
| SPC_3146 | SCPS53 | *-* | putative mannitol dehydrogenase |
| SPC_3459 | SCPS56 | *-* | putative membrane domain protein involved in signal transduction |
| SPC_3681 | SCPS61 | *yhjC* | putative transcriptional regulator |
| SPC_3744 | SC3599 | *malS* | periplasmic alpha-amylase precursor |
| SPC_3911 | SCPS68 | *torT* | periplasmic sensor in multi-comopnent regulatory system with TorS (sensory kinase) and TorR (regulator) |
| SPC_3932 | SCPS69 | *-* | putative LysR family transcriptional regulator |
| SPC_3943 | SCPS70 | *-* | putative phosphotransferase system fructose-specific component IIB |
| SPC_3988 | SCPS82 | *-* | putative cytoplasmic protein |
| SPC_4052 | SC3843_SC3844 | *-* | possible exported protein |
| SPC_4074 | SCPS146 | *-* | putative arylsulfatase regulator |
| SPC_4140 | SCPS76 | *fdoG* | formate dehydrogenase |
| SPC_4348 | SCPS86 | *fdh* | formate dehydrogenase |
| SPC_4518 | SCPS122 | *-* | putative phage shock protein A |
| SPC_4632 | 4661198_4661470 bp | *-* | hypothetical protein |
| SPC_4652 | 4676818_4676442 bp | *-* | hypothetical protein |
| SPC_4660 | SCPS123 | *-* | putative NAD-dependent aldehyde dehydrogenase |
| SPC_4686 | SCPS150 | *-* | putative LuxR/UhpA family transcriptional regulator |
| SPC_4730 | SC4442 | *-* | putative periplasmic protein |

(B) List of genes that are inactivated in *S. paratyphi* C but normal in *S. choleraesuis*.

| *S. paratyphi* C | *S. choleraesuis* | symbol | product |
| --- | --- | --- | --- |
| SPC_0165 | SC0153 | *lpdA* | lipoamide dehydrogenase |
| SPC_0213 | SC0197 | *stfD* | putative periplasmic fimbrial chaperone |
| SPC_0252 | SC0236 | *mesJ* | cell cycle protein |
| SPC_0311 | SC0294 | *safC* | putative fimbriae usher |
| SPC_0357 | SC0389 | *-* | possible outer membrane protein |
| SPC_0381 | SC0412 | *prpE* | PrpE protein |
| SPC_0440 | SC0469 | *-* | 2-aminoethylphosphonate transporter,ATPase component |
| SPC_0460 | SC0488 | *-* | morphogene; putative regulator of murein genes (BolA family) |
| SPC_0473 | SC0501 | *ybaO* | hypothetical transcriptional regulator |
| SPC_0563 | SC0588 | *fimZ* | probable transcriptional regulator |
| SPC_0581 | SC0600 | *-* | putative transcriptional regulator (AraC/XylS family) |
| SPC_0673 | SC0685 | *ybeS* | putative molecular chaperone, DnaJ family |
| SPC_0797 | SC0798 | *slrP* | leucine-rich repeat protein |
| SPC_0860 | SC0855 | *-* | putative dehydrogenase |
| SPC_0911 | SC0866 | *-* | glutaredoxin-1 homolog |
| SPC_0943 | SC0896 | *ybjY* | hypothetical protein |
| SPC_1179 | SC2476 | *acrD* | RND family aminoglycoside/multidrug efflux pump |
| SPC_1265 | SC0326 | *-* | Eaa protein |
| SPC_1333 | SC2374 | *-* | hypothetical protein |
| SPC_1396 | SC2315 | *yfbK* | putative von Willebrand factor, vWF type A domain |
| SPC_1439 | SC2276 | *-* | putative dehydratase protein |
| SPC_1447 | SC2268 | *alkB* | DNA repair system protein |
| SPC_1504 | SC2214 | *-* | putative regulatory protein |
| SPC_1510 | SC2208 | *-* | putative inner membrane protein |
| SPC_1511 | SC2207 | *galS* | GalR/LacI family transcriptional repressor of mgl operon |
| SPC_1531 | SC2188 | *yohG* | putative lipoprotein |
| SPC_1553 | SC2165 | *-* | putative outer membrane protein |
| SPC_1561 | SC2157 | *-* | putative MFS family transport protein |
| SPC_1564 | SC2154 | *-* | hypothetical protein |
| SPC_1715 | SC2003 | *yeeI* | hypothetical protein |
| SPC_1751 | SC1967 | *amyA* | cytoplasmic alpha-amylase |
| SPC_1757 | SC1961 | *fliB* | N-methylation of lysine residues in flagellin |
| SPC_1772 | SC1945 | *-* | putative cell wall-associated hydrolase |
| SPC_1805 | SC1915 | *yecM* | putative cytoplasmic protein |
| SPC_1894 | SC1829 | *rrmA* | 23S rRNA m1G745 methyltransferase |
| SPC_1925 | SC1797 | *-* | putative cytoplasmic protein |
| SPC_2034 | SC1691 | *-* | putative inner membrane protein |
| SPC_2056 | SC1670 | *-* | putative aldo/keto reductase family |
| SPC_2077 | SC1652 | *-* | putative chemo-receptor protein |
| SPC_2146 | SC1586 | *-* | putative NADP-dependent oxidoreductase |
| SPC_2162 | SC1576 | *narU* | MFS superfamily, nitrate extrusion protein |
| SPC_2203 | SC1542 | *yneH* | glutaminase |
| SPC_2211 | SC1535 | *ydeD* | putative permease |
| SPC_2222 | SC1524 | *-* | putative membrane transport protein |
| SPC_2265 | SC1483 | *-* | putative periplasmic protein |
| SPC_2378 | SC1371 | *ydiS* | flavoprotein |
| SPC_2387 | SC1363 | *-* | putative Diguanylate cyclase/phosphodiesterase domain 1 |
| SPC_2400 | SC1351 | *-* | putative DNA/RNA non-specific endonuclease |
| SPC_2421 | SC1330 | *-* | hypothetical protein |
| SPC_2425 | SC1327 | *astB* | succinylarginine dihydrolase |
| SPC_2427 | SC1327 | *astA* | arginine succinyltransferase |
| SPC_2458 | SC1295 | *-* | putative Methyl-accepting chemotaxis protein; Diguanylate cyclase/phosphodiesterase domain 1 |
| SPC_2463 | SC1289 | *yeaN* | hypothetical protein |
| SPC_2542 | SC1154 | *fhuE* | outer membrane receptor for Fe(III)-coprogen, Fe(III)-ferrioxamine B and Fe(III)-rhodotrulic acid uptake |
| SPC_2597 | SC1100 | *msyB* | acidic protein suppresses mutants lacking function of protein export |
| SPC_2618 | SC1081 | *-* | putative secreted protein |
| SPC_2780 | SC2672 | *-* | 3-deoxy-7-phosphoheptulonate synthase |
| SPC_2791 | SC2681 | *-* | putative cytoplasmic protein |
| SPC_2809 | SC2701 | *fljA* | Flagellar synthesis: repressor of fliC |
| SPC_2853 | SC2741 | *-* | ribonucleotide-diphosphate reductase alpha subunit |
| SPC_2865 | SC2754 | *yqaA* | putative inner membrane protein |
| SPC_2881 | SC2773 | *-* | anaerobic nitric oxide reductase flavorubredoxin |
| SPC_2964 | SC2852 | *-* | putative 3-polyprenyl-4-hydroxybenzoate decarboxylase |
| SPC_2991 | SC2877 | *-* | phosphoadenosine phosphosulfate reductase |
| SPC_3019 | SC2901 | *-* | putative MFS superfamily D-glucarate permease |
| SPC_3027 | SC2909 | *-* | putative HAAAP family, serine transport protein |
| SPC_3196 | SC3069 | *-* | putative NAD-dependent aldehyde dehydrogenase |
| SPC_3533 | SC3398 | *prkB* | phosphoribulokinase |
| SPC_3591 | SC3453 | *rtcR* | sigma N (sigma 54)-dependent regulator of rtcBA expression |
| SPC_3628 | SC3489 | *livF* | high-affinity branched-chain amino acid transporter |
| SPC_3689 | SC3548 | *dctA* | DAACS family C4-dicarboxylic acids transport protein |
| SPC_3723 | SC3576 | *bisC* | biotin sulfoxide reductase |
| SPC_3779 | SC3621 | *-* | putative mandelate racemase |
| SPC_3812 | SC3653 | *-* | phosphopantothenoylcysteine synthase/decarboxylase |
| SPC_3831 | SC3673 | *yicI* | putative alpha-xylosidase |
| SPC_3866 | SC3706 | *-* | putative regulatory protein, gntR family |
| SPC_3909 | SC3741 | *-* | trimethylamine N-oxide reductase |
| SPC_4172 | SC3956 | *-* | putative permease of the Na+:galactoside symporter family |
| SPC_4299 | SC4117 | *-* | DNA-damage-inducible protein F, induced by UV and mitomycin C; SOS, lexA regulon |
| SPC_4338 | SC4153 | *-* | hypothetical protein |
| SPC_4359 | SC4175 | *adi* | arginine decarboxylase |
| SPC_4385 | SC4197 | *phoN* | nonspecific acid phosphatase precursor |
| SPC_4505 | SC4234 | *-* | N-acetylmuramoyl-l-alanine amidase II, a murein hydrolase |
| SPC_4589 | SC4312 | *-* | P-type ATPase, Mg2+ ATPase transporter |
| SPC_4634 | SC4356 | *yjhP* | putative SAM-dependent methyltransferase |
| SPC_4649 | SC4367 | *-* | isoaspartyl dipeptidase |
| SPC_4723 | SC4435 | *-* | tolerance to colicin E2 |

(C) List of genes that are inactivated in *S. choleraesuis* but normal in *S. paratyphi* C.

| *S. paratyphi* C | *S. choleraesuis* | symbol | product |
| --- | --- | --- | --- |
| SPC_0044 | SCPS87 | *-* | putative transport protein |
| SPC_0110 | SCPS9 | *araB* | ribulokinase |
| SPC_0124 | SCPS15 | *leuO* | leucine transcriptional activator |
| SPC_0257 | SCPS46 | *cutF* | copper homeostasis protein CutF precursor |
| SPC_0282 | SCPS50 | *-* | ClpB-like protein |
| SPC_0347 | SCPS73 | *stbE* | fimbrial chaperone protein |
| SPC_0362 | SCPS77 | *-* | putative cation efflux system protein |
| SPC_0364 | SCPS79 | *-* | putative cation transport ATPase |
| SPC_0491 | SCPS90 | *acrR* | potential acrAB operon repressor |
| SPC_0512 | SCPS91 | *-* | probable secreted protein |
| SPC_0532 | SCPS92 | *gcl* | glyoxylate carboligase |
| SPC_0559 | SCPS2 | *fimC* | fimbrial chaperone protein |
| SPC_0565 | SCPS132 | *-* | hypothetical protein |
| SPC_0628 | SCPS96 | *ybdR* | hypothetical zinc-dependant alcohol dehydrogenase |
| SPC_0643 | SCPS98 | *pagP* | antimicrobial peptide resistance and lipid A acylation protein |
| SPC_0667 | SCPS99 | *-* | putative hydrolase C-terminus |
| SPC_0699 | SCPS101 | *citB* | citrate utilization protein B |
| SPC_0718 | SCPS133 | *ybfA* | hypothetical protein |
| SPC_0728 | SCPS154 | *-* | hypothetical protein |
| SPC_0807 | SCPS135 | *-* | putative inner membrane protein |
| SPC_0851 | SCPS4 | *-* | hypothetical protein |
| SPC_0913 | SCPS6 | *nfsA* | oxygen-insensitive NADPH nitroreductase |
| SPC_0995 | SCPS102 | *mukB* | condesin subunit B |
| SPC_1011 | SCPS7 | *-* | exodeoxyribonuclease |
| SPC_1112 | SCPS45 | *hscA* | chaperone protein HscA |
| SPC_1122 | SCPS44 | *-* | putative lipoprotein |
| SPC_1123 | SCPS43 | *pbpC* | penicillin-binding protein 1C |
| SPC_1142 | SCPS112 | *shdA* | AIDA autotransporter-like protein |
| SPC_1180 | SCPS42 | *narQ* | nitrate/nitrite sensor protein NarQ |
| SPC_1200 | SCPS111 | *eutA* | putative ethanolamine utilization protein EutA |
| SPC_1202 | SCPS41 | *eutC* | ethanolamine ammonia-lyase small subunit |
| SPC_1204 | SCPS40 | *eutK* | ethanolamine utilization protein EutK |
| SPC_1407 | SCPS142 | *pmrD* | polymyxin B resistance protein |
| SPC_1412 | SCPS39 | *yfbG* | hypothetical protein |
| SPC_1417 | SCPS109 | *-* | hypothetical protein |
| SPC_1470 | SCPS156 | *sspH2* | Leucine-rich repeat protein |
| SPC_1508 | SCPS37 | *yeiG* | putative esterase |
| SPC_1578 | SCPS128 | *-* | hypothetical protein |
| SPC_1659 | 2152106_2152292 bp | *pduU* | putative propanediol utilization protein PduU |
| SPC_1773 | SCPS108 | *-* | putative glucose-6-phosphate dehydrogenase |
| SPC_1849 | SCPS127 | *-* | hypothetical protein |
| SPC_1937 | SCPS140 | *appB* | probable cytochrome oxidase subunit II |
| SPC_1938 | SCPS105 | *-* | putative cytochrome oxidase subunit I |
| SPC_1943 | SCPS32 | *-* | hydrogenase-1 large chain (nifE hydrogenase) |
| SPC_2064 | SCPS30 | *-* | invasin-like protein |
| SPC_2066 | SCPS29 | *-* | putative thiol peroxidase |
| SPC_2096 | SCPS104 | *cybB* | cytochrome b561 |
| SPC_2112 | SCPS27 | *-* | putative esterase |
| SPC_2125 | SCPS26 | *ydcK* | putative transferase |
| SPC_2161 | SCPS24 | *narZ* | respiratory nitrate reductase 2 alpha chain |
| SPC_2205 | SCPS20 | *-* | putative regulatory protein |
| SPC_2239 | SCPS19 | *-* | putative voltage-gated ClC-type chloride channel ClcB |
| SPC_2269 | SCPS17 | *-* | putative oxidoreductase |
| SPC_2580 | SCPS14 | *mviM* | putative virulence factor MviM |
| SPC_2617 | SCPS13 | *-* | putative sialic acid transporter |
| SPC_2621 | SCPS126 | *-* | putative sodium/glucose cotransporter |
| SPC_2635 | SCPS12 | *scsB* | membrane protein, suppressor for copper-sensitivity B precursor |
| SPC_2687 | SCPS11 | *uup* | ABC transporter ATP-binding protein |
| SPC_2720 | SCPS8 | *-* | hypothetical protein |
| SPC_2783 | SCPS47 | *yfiN* | hypothetical protein |
| SPC_2810 | SCPS48 | *fljB* | flagellar biosynthesis protein |
| SPC_2953 | SCPS49 | *-* | possible membrane transport protein |
| SPC_2954 | SCPS21 | *-* | possible LysR-family transcriptional regulator |
| SPC_2987 | SCPS51 | *-* | putative cytoplasmic protein |
| SPC_2988 | SCPS114 | *ygcB* | putative helicase |
| SPC_3083 | SCPS143 | *yohM* | putative inner membrane protein |
| SPC_3309 | SCPS54 | *-* | hypothetical protein |
| SPC_3326 | SCPS116 | *garD* | D-galactarate dehydratase |
| SPC_3363 | SCPS144 | *folP* | dihydropteroate synthase |
| SPC_3462 | SCPS57 | *acrE* | transmembrane protein affecting septum formation and cell membrane permeability |
| SPC_3548 | SCPS129 | *bigA* | putative surface-exposed virulence protein |
| SPC_3600 | SCPS59 | *-* | putative dihydroxyacid dehydratase |
| SPC_3618 | SCPS145 | *-* | putative phosphotriesterase |
| SPC_3632 | SCPS60 | *livK* | leucine-specific binding protein |
| SPC_3716 | SCPS1 | *lpfC* | long polar fimbrial outer membrane usher protein |
| SPC_3757 | SCPS62 | *yiaS* | L-ribulose-5-phosphate 4-epimerase |
| SPC_3759 | SCPS63 | *-* | hypothetical protein |
| SPC_3780 | SCPS64 | *-* | putative permease |
| SPC_3847 | SCPS118 | *-* | putative transferase |
| SPC_3855 | SCPS65 | *yicM* | putative inner membrane transport protein |
| SPC_3869 | SCPS66 | *uhpC* | regulatory protein |
| SPC_3898 | SCPS119 | *ccmH* | putative heme lyase subunit |
| SPC_3948 | SCPS71 | *-* | hypothetical protein |
| SPC_4007 | SCPS83 | *purH* | bifunctionalphosphoribosylaminoimidazolecarboxamide formyltransferase/IMP cyclohydrolase |
| SPC_4014 | SCPS72 | *ilvG* | acetolactate synthase II large subunit |
| SPC_4093 | SCPS74 | *trkH* | trk system potassium uptake protein |
| SPC_4101 | SCPS75 | *yihG* | putative acyltransferase |
| SPC_4124 | SCPS120 | *yihS* | putative isomerase |
| SPC_4180 | SCPS78 | *ego* | putative ABC transporter ATP-binding protein |
| SPC_4214 | SCPS80 | *-* | hypothetical protein |
| SPC_4223 | SCPS81 | *pflD* | putative pyruvate formate lyase II |
| SPC_4249 | SCPS84 | *yjbB* | hypothetical protein |
| SPC_4264 | SCPS147 | *-* | putative inner membrane protein |
| SPC_4271 | SCPS85 | *-* | putative phage tail protein |
| SPC_4365 | SCPS155 | *-* | hypothetical protein |
| SPC_4523 | SCPS88 | *yjfC* | hypothetical protein |
| SPC_4550 | SCPS89 | *ytfG* | hypothetical protein |
| SPC_4564 | SCPS148 | *-* | dihydroorotase |
| SPC_4619 | SCPS130 | *-* | integrase |
| SPC_4646 | SCPS149 | *-* | putative cytoplasmic protein |
| SPC_4672 | SCPS124 | *-* | putative transcriptional regulator |
